# Oral Bacteria and Cancer

**DOI:** 10.1371/journal.ppat.1003933

**Published:** 2014-03-27

**Authors:** Sarah E. Whitmore, Richard J. Lamont

**Affiliations:** Center for Oral Health and Systemic Disease, School of Dentistry, University of Louisville, Louisville, Kentucky, United States of America; The University of North Carolina at Chapel Hill, United States of America

## Epidemiological Associations

Over a number of years, epidemiological studies established several well-defined risk factors for cancer, including age, heredity, diet, tobacco use, chronic viral infections, and inflammation. Paradoxically, the success of these studies left little room for incorporation of any new factors or causative agents, and, consequently, the idea that a bacterial infection could contribute to cancer was generally disregarded. However, landmark studies in the early 1990s established *Helicobacter pylori* as a causative agent of gastric cancers, resulting in a paradigm shift regarding the relationship between microbial agents and cancers [1]. Indeed, in 1994, *H. pylori* became the first bacterial species to be officially recognized by the World Health Organization as a definite cause of cancer in humans. Since then, there has been a growing body of evidence supporting an association between specific microorganisms, including those in the oral cavity, and various types of cancers.

The oral cavity is inhabited by complex multispecies communities that usually exist in a balanced immunoinflammatory state with the host [2]. Certain species, such as *Porphyromonas gingivalis*, can disrupt this equilibrium, resulting in a dysbiotic host–microbiota interaction. Subsequently, other community constituents, such as *Fusobacterium nucleatum*, can become opportunistically pathogenic, and the combined effect of a dysbiotic microbial community along with a dysregulated immune response ultimately causes periodontal disease [2]. These well-studied periodontal organisms have now emerged as the focal point for the developing association between oral bacteria and cancer.

Perhaps the most likely carcinogenic link with oral bacteria is with oral squamous cell carcinoma (OSCC), one of the most common cancers worldwide. OSCC surfaces have been reported to harbor significantly higher levels of *Porphyromonas* and *Fusobacterium* compared with contiguous healthy mucosa [3]. Moreover, immunohistochemistry with *P. gingivalis* antibodies revealed higher levels of detection and intensity of staining in gingival carcinomas compared with healthy gingival tissue, although only a small number of cases were examined [4]. A striking association has also been demonstrated between *P. gingivalis* infection and pancreatic cancer. In a prospective cohort study of over 400 cases and controls, a >2-fold increase in risk of pancreatic cancer was observed among those with high levels of antibodies to *P. gingivalis*, after adjusting for known risk factors [5]. Similarly, in the extensive National Health and Nutrition Examination Survey III, orodigestive cancer mortality was found to be related to the levels of *P. gingivalis* antibodies, independent of periodontal disease [6]. Several recent studies have shown a strong association between *F. nucleatum* and colorectal cancer (CRC) [7]–[10]. *F. nucleatum* was found to be one of the more abundant species within and around CRC neoplasms, and levels of *F. nucleatum* correlated with the presence of lymph node metastases.

## Mechanistic Basis Supporting a Role for Oral Bacteria in Cancer

Epidemiological studies associate oral bacteria temporally and spatially with certain cancers and render involvement in the initiation or progression of the disease plausible. However, it is equally plausible that early undetected cancer, or precancerous lesions, facilitate the colonization and growth of oral bacteria. If these organisms are active participants in the disease process, then a mechanistic basis that would support an etiological role should exist.

Chronic or dysregulated inflammation has long been appreciated as contributing to tumor development, in part through modulation of the tumor microenvironment [11]. Both *P. gingivalis* and *F. nucleatum* establish chronic infections that involve intracellular persistence within epithelial cells, can spread systemically and cause extra-oral infections, and have well-characterized immune disruptive properties [12]. *F. nucleatum* is strongly proinflammatory, and McCoy et al. [7] demonstrated a positive correlation between mRNA levels for several local cytokines and *Fusobacterium* species in CRC cases. Furthermore, in the *Apc^Min/+^* mouse model of intestinal tumorigenesis, *F. nucleatum* recruits tumor-infiltrating immune cells, thus generating a proinflammatory microenvironment that is conducive for CRC progression [13]. The inflammatory properties of *P. gingivalis* are more nuanced, and the organism can exhibit both pro- and anti-inflammatory properties, depending on the context [14], [15]. In either event, *P. gingivalis* has a major disruptive effect on local immune responses in the periodontal area [2]; however, the possible link with tumor development has yet to be investigated in molecular detail. In addition to broadly based immune-disruptive properties, both *P. gingivalis* and *F. nucleatum* impinge upon several aspects of epithelial cell signaling that have relevance to cancer progression.

### *P. gingivalis*

**Figure 1:**
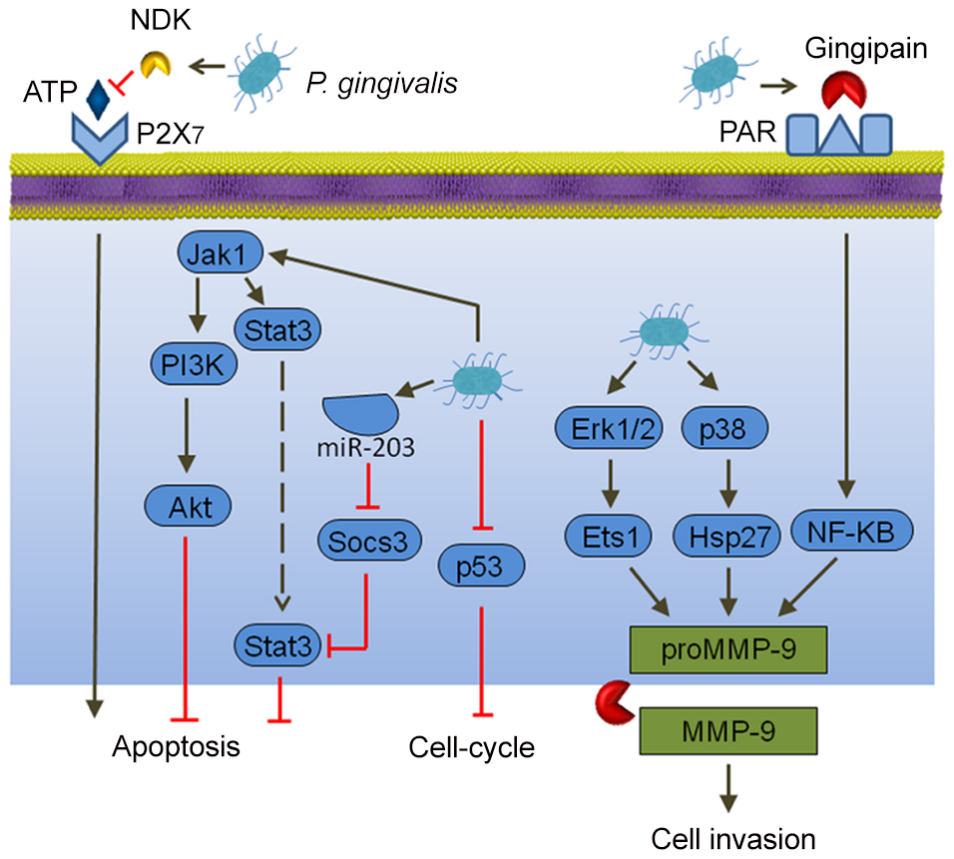
Interactions between *P. gingivalis* and epithelial cells that could produce an oncogenic phenotype. Extracellular *P. gingivalis* secrete gingipains, which activate Protease Activated Receptor (PAR) leading to promatrix metalloprotease (MMP)-9 production, and they also convert proMMP-9 to mature MMP-9, along with nucleoside diphosphate kinase (NDK), which cleaves ATP and prevents activation of the proapoptotic P2X_7_ receptor. Intracellular *P. gingivalis* activate antiapoptotic Jak-Stat signaling and inhibit expression of the p53 tumor suppressor. Additionally, Erk 1/2 and p38 are activated, which also elevates proMMP-9 expression.

Cancer cells, by definition, are defective in functional cell death pathways, and tumorigenesis is initiated when cells are freed from growth restraints. Epithelial cell responses to *P. gingivalis* infection include both changes to apoptosis and cell division (Figure 1). In primary cultures of gingival epithelial cells, *P. gingivalis* is strongly antiapoptotic and, indeed, can suppress chemically induced apoptosis [16]. *P. gingivalis* activates Jak1/Akt/Stat3 signaling that controls intrinsic mitochondrial apoptosis pathways [16], [17]. At the mitochondrial membrane, the activity of proapoptotic Bad is inhibited, and the Bcl2 (antiapoptotic):Bax (proapoptotic) ratio is increased, consequently curtailing the release of the apoptosis effector cytochrome c [18]. Further downstream, activation of both caspase-9 and the executioner caspase-3 is blocked. Remarkably, *P. gingivalis* possesses multiple mechanisms for inhibition of apoptosis in epithelial cells. Expression of microRNAs (miRs) is modulated, and up-regulation of miR-203 leads to inhibition of the negative regulator SOCS3 and subsequent suppression of apoptosis [19]. *P. gingivalis* secretes a nucleoside diphosphate kinase (NDK), which can function as an ATPase and prevent ATP-dependent apoptosis mediated through the purinergic receptor P2X_7_ [20]. Another potential role for NDK is diminishing ATP activation of P2X_7_ receptors on dendritic cells, which will impede activation of the NLRP3/ASC/caspase-1 inflammasome. This, in turn, will reduce secretion of IL-1β, which is important for the priming of IFNγ-producing tumor–antigen-specific CD8^+^ T cells [21]. In concert with suppression of apoptosis, *P. gingivalis* can accelerate progression through the S-phase of the cell cycle by manipulation of cyclin/CDK (cyclin-dependent kinase) activity and reducing the level of the p53 tumor suppressor [22]. A fimbrial-deficient mutant of *P. gingivalis* does not display this activity, suggestive of a role for the FimA adhesin in elevating epithelial cell proliferation. A role for LPS (lipopolysaccharide) in the dysregulation of p53 has been established [23], and the extent to which *P. gingivalis* LPS can target p53 requires further investigation.

In both primary gingival epithelial cells and OSCC cells, *P. gingivalis* can induce the expression of the B7-H1 and B7-DC receptors [24]. These receptors are up-regulated in cells originating from a variety of cancers and contribute to chronic inflammation. Furthermore, B7-H1 expression promotes the development of regulatory T cells (Treg), which suppress effector T cells, and thus could contribute to immune evasion by oral cancers. Another impact of *P. gingivalis* on OSCC cells is in promoting cellular invasion. *P. gingivalis* infection activates the ERK1/2-Ets1, p38/HSP27, and PAR2/NF-ΚB pathways to induce promatrix metalloproteinase (MMP)-9 expression [25]. Gingipains, cysteine proteinases produced by *P. gingivalis*, play a dual role in this process. They both engage the PAR2 receptor and cleave the MMP-9 proenzyme into the mature active form. MMP-9 degrades basement membrane and extracellular matrix, which promotes carcinoma cell migration and invasion, thus allowing carcinoma cells to enter the lymphatic system and blood vessels for dissemination and metastatic growth at remote sites. In this manner, *P. gingivalis* may contribute to OSCC metastasis.

### *F. nucleatum*

**Figure 2:**
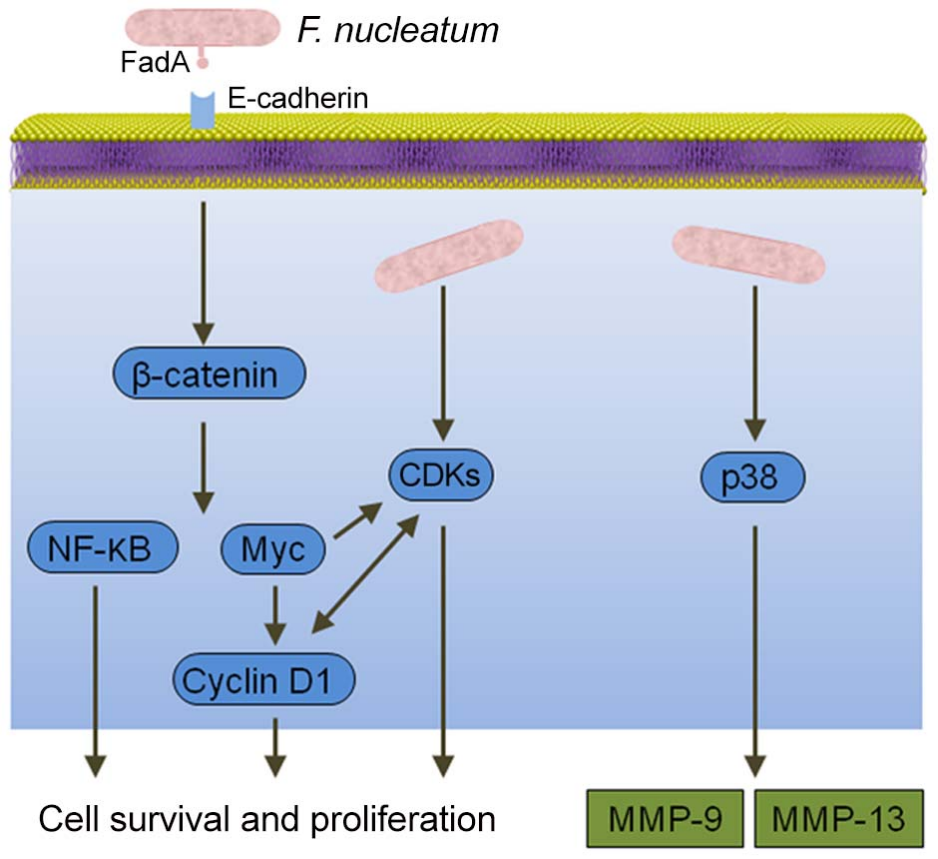
Interactions between *F. nucleatum* and epithelial cells that could produce an oncogenic phenotype. Binding of the FadA adhesin to E-cadherin activates β-catenin signaling, resulting in activation of genes that control cell survival and proliferation. *F. nucleatum* also activates several cyclin dependent kinases (CDKs) and p38, which controls the production of matrix metalloproteases MMP-9 and MMP-13.

Epithelial cell responses to *F. nucleatum* infection are also consistent with carcinogenesis (Figure 2). Signaling molecules targeted by *F. nucleatum* include kinases involved in cell cycle control, and, as a result, *F. nucleatum* can elevate cell proliferation and migration [26]. *F. nucleatum* also activates p38, leading to the secretion of MMP-9 and MMP-13 (collagenase 3). Similar to MMP-9, MMP-13 plays an important role in tumor invasion and metastasis. Recently, a more direct relationship between *F. nucleatum* and CRC was demonstrated whereby the fusobacterial adhesin FadA binds to E-cadherin on colon cancer cells and activates β-catenin signaling [27]. This pathway leads to increased transcriptional activity of oncogenes, Wnt, and pro-inflammatory cytokines, as well as stimulation of CRC cell proliferation. In vivo relevance was established by the finding that *fadA* gene levels in colon tissue from patients with CRC were >10-fold higher compared with normal individuals.

## Conclusions

Both *P. gingivalis* and *F. nucleatum* have attributes consistent with a role in cancer development and progression. The question then arises as to why the widespread infection with these organisms leads to disease in only a limited number of individuals. Part of the answer may relate to the community nature of oral infections and the potential constraining influence of other bacteria. However, another consideration is the multifactorial etiology of cancer, and, within this framework, specific oral bacteria and their associated inflammatory insults may play a contributory, but not exclusive, role.

The implications of oral bacterial involvement in cancer are many. The detection of *P. gingivalis* or *F. nucleatum* in precancerous lesions could be used as a poor prognosis indicator. Improved oral hygiene and treatment of periodontitis may be useful in limiting the development or spread of cancer. Finally, since well-characterized virulence factors of *P. gingivalis* and *F. nucleatum*, such as the FimA and FadA adhesins, may function as effector molecules in the transition of normal epithelial cells to cancerous cells, they may provide novel targets for therapeutic intervention.
